# GABA Receptors Can Depolarize the Neuronal Membrane Potential via Quantum Tunneling of Chloride Ions: A Quantum Mathematical Study

**DOI:** 10.3390/cells11071145

**Published:** 2022-03-28

**Authors:** Sager Nawafleh, Abdallah Barjas Qaswal, Aiman Suleiman, Obada Alali, Fuad Mohammed Zayed, Mohammad Abu Orabi Al-Adwan, Mo’ath Bani Ali

**Affiliations:** 1 Department of Anesthesia and Intensive Care Unit, The Hashemite University, Zarqa 13115, Jordan; 2 School of Medicine, The University of Jordan, Amman 11942, Jordan; fuad.41994@gmail.com (F.M.Z.); dr.mohaladwan@hotmail.com (M.A.O.A.-A.); moathmegdadi@gmail.com (M.B.A.); 3 Department of Anesthesia, Intensive Care and Pain Management, Harvard Medical School, Beth Israel Deaconess Medical Center, Boston, MA 02215, USA; asuleima@bidmc.harvard.edu; 4 Department of Anesthesia and Intensive Care, Alabdali Clemenceau Hospital, Amman 11190, Jordan; alaliobada.ane@gmail.com

**Keywords:** GABA receptors, chloride ions, membrane potential, depolarization, hyperpolarization, quantum tunneling, quantum conductance, quantum biology, quantum medicine

## Abstract

GABA (gamma-aminobutyric acid) receptors represent the major inhibitory receptors in the nervous system and their inhibitory effects are mediated by the influx of chloride ions that tends to hyperpolarize the resting membrane potential. However, GABA receptors can depolarize the resting membrane potential and thus can also show excitatory effects in neurons. The major mechanism behind this depolarization is mainly attributed to the accumulation of chloride ions in the intracellular compartment. This accumulation leads to increase in the intracellular chloride concentration and depolarize the Nernst potential of chloride ions. When the membrane potential is relatively hyperpolarized, this will result in a chloride efflux instead of influx trying to reach their depolarized equilibrium potential. Here, we propose different mechanism based on a major consequence of quantum mechanics, which is quantum tunneling. The quantum tunneling model of ions is applied on GABA receptors and their corresponding chloride ions to show how chloride ions can depolarize the resting membrane potential. The quantum model states that intracellular chloride ions have higher quantum tunneling probability than extracellular chloride ions. This is attributed to the discrepancy in the kinetic energy between them. At physiological parameters, the quantum tunneling is negligible to the degree that chloride ions cannot depolarize the membrane potential. Under certain conditions such as early neuronal development, gain-of-function mutations, stroke and trauma that can lower the energy barrier of the closed gate of GABA receptors, the quantum tunneling is enhanced so that the chloride ions can depolarize the resting membrane potential. The major unique feature of the quantum tunneling mechanism is that the net efflux of chloride ions is attained without the need for intracellular accumulation of chloride ions as long as the energy barrier of the gate is reduced but still higher than the kinetic energy of the chloride ion as a condition for quantum tunneling to take place.

## 1. Introduction

GABA (gamma-aminobutyric acid) receptors are pentameric ligand-gated receptors and are considered to be inhibitory receptors because they hyperpolarize the resting membrane potential by the influx of chloride ions. However, GABA receptors can depolarize the membrane potential if there is an intracellular chloride accumulation that increases the intracellular concentration and makes its Nernst potential less negative. Accordingly, when the chloride’s Nernst potential becomes less negative than the resting membrane potential, the opening of GABA receptors will result in chloride efflux instead of chloride influx. This chloride efflux will result in membrane depolarization instead of hyperpolarization [1,2].

The action of depolarization mediated by GABA receptors occurs physiologically during early development or pathologically in certain conditions such as trauma, stroke, and epilepsy [1,3,4,5,6]. The main mechanism behind this depolarization is the increase in the intracellular chloride ions, which makes the Nernst potential less negative [7,8]. The molecular mechanism behind this alteration in the homeostasis of chloride ions concentrations is the abnormal expression of NKCC1 transporter, which loads the chloride ions inside neurons, and KCC2 transporter, which extrudes chloride ions outside the neurons [4,5,6,8]. The higher expression of NKCC1 and the lower expression of KCC2 leads to increased intracellular chloride ions concentration, which results in membrane depolarization mediated by chloride ions efflux [4,5,6,8].

However, because the intracellular chloride concentrations cannot be reliably measured depending on the expression levels of chloride transporters and the estimation of the intracellular concentration is not accurate since the in-vitro studies do not take into consideration the neuronal activity, the actual intracellular chloride concentration may be not that high to induce membrane depolarization in the corresponding conditions. Hence, an alternative mechanism may be required to explain the depolarization action mediated by GABA receptors [8].

The model of quantum tunneling of ions has been studied previously in hypothetical biological frameworks and was found to generate solid mathematical solutions on atomic and molecular levels in nascent fields of neurophysiology and pharmacokinetics [9,10,11].

In our paper, we present a different explanation of the depolarization induced by GABA receptors based on the model of quantum tunneling of chloride ions through the closed gate. The quantum model will be able to explain the depolarization action of the GABA receptors without the requirement of intracellular chloride accumulation. This will provide an alternative mechanism of depolarization in case the increase in the intracellular chloride concentration is not sufficient to depolarize the membrane potential. Moreover, specific features of the quantum model will be presented to exhibit its distinction over the classical model. Moreover, as the action of depolarization in the context of GABA receptor’s function can result in either excitation or inhibition according to a recent study [8], we do not aim to explore this idea in the context of the quantum model, but we aim to focus on the action of depolarization itself. However, we will propose certain mechanisms by which the quantum model can be exploited in such a way that can be tested experimentally and be used to explain the physiological and pathophysiological effects of depolarization induced by GABA receptors.

## 2. Materials and Methods

### The Mathematical Model of Chloride Ions Quantum Tunneling

The model of quantum tunneling of ions has been applied on the voltage-gated channels [12,13]. Herein, we apply the quantum model on the closed GABA receptors and chloride ions. This is valid because the voltage-gated channel and the GABA receptor possess a closed hydrophobic gate that blocks the permeation of ions [14,15].

The quantum tunneling probability of chloride ions through the closed gate of GABA receptors can be calculated by the following equation [12,13]:

(1) $$T_{Q}=e^{\frac{-\sqrt{8m}}{\hbar }\times \frac{2w}{3g}\sqrt{{\left(g-KE\right)}^{3}}}$$

where $T_{Q}$ is the probability of tunneling, *m* is the mass of chloride ion (5.9 × 10^−26^ kg), *g* is the height of the energy barrier of the closed gate in the GABA receptor or simply ‘the energy of the gate’, *w* is the length of the gate, and *KE* is the kinetic energy of chloride ion.

**Figure 1:**
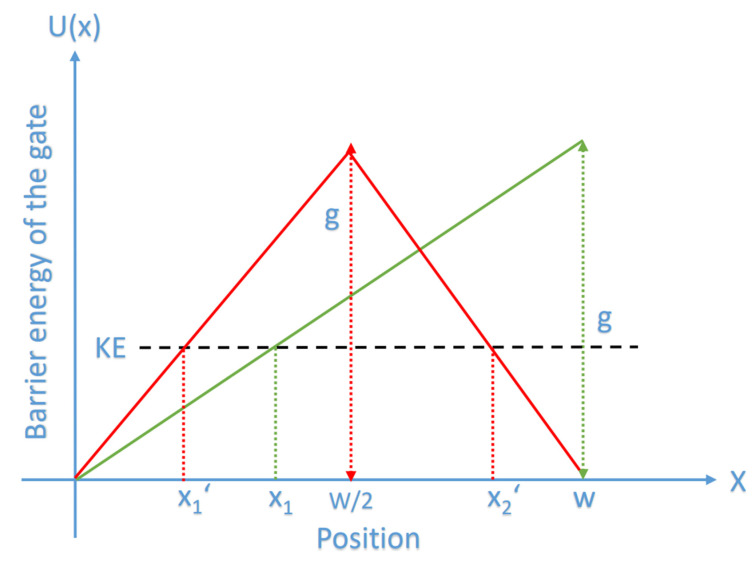
The figure represents a schematic diagram of two possible shapes of the energy barrier of the gate with respect to the ion’s position *x* through the gate. The green curve represents a triangle energy barrier in which the energy increases linearly till the end of the gate reaching the maximum energy value *g* similar to the energy barrier of the regular electric field, while the red curve represents a triangle barrier that shows a symmetry at the center similar to what observed experimentally in the hydrophobic materials. The red curve describes a linear increase in the energy of the gate till reaching the center where it reaches the maximum value *g* and then it starts to decrease by the same rate till the end of the gate. *x*_1_ is where the kinetic energy of the ion equals the barrier energy at the green curve, while *x*_1_′ and *x*_2_′ are the ion’s positions where the kinetic energy of the ion equals the barrier energy on the red curve.

Equation (1) has been applied before on the closed gate of the voltage-gated channels, and in the present study, we are going to apply the model of quantum tunneling of chloride ions on the closed gate of the GABA receptor. The hydrophobic gating in voltage-gated channels and pentameric ligand-gated channels, including GABA receptors has been shown to be a reasonable mechanism that controls the flow of ions [14,15,16,17,18]. The hydrophobic gate forms an energy barrier higher than the kinetic energy of the passing ions [14,15,16,17,18]. This gate is formed by hydrophobic residues that come close to each other to form a narrow pore. In the previous studies, the closed gate was viewed as barrier similar to barrier shape of a regular electric field [12,13]. This means that the energy barrier takes the shape of a triangle, see Figure 1. The experimental evidence showed that the energy barrier for the ion while passing through a hydrophobic material, which includes a hydrophobic membrane or a hydrophobic gate in ion channels, changes in a shape similar to the shape of the barrier of a regular electric field or the shape of a triangle [17,18,19,20,21,22,23], which is represented by two possible triangle shapes in Figure 1 that are indicated by the green and red curves. We don’t say that the hydrophobic gate is a regular electric field, rather we say that the barrier shape of the gate can be viewed as the barrier shape of a regular electric field based on the experimental observations [17,18,19,20,21,22,23]. The curves in Figure 1 may not reflect the exact relationship between the energy barrier and the ion’s position through the gate, but they provide a reasonable approximation of the barrier shape based on the experimental results [17,18,19,20,21,22,23]. This approximation is rational to be made because there is no clear mathematical equation that can describe how the energy barrier changes with respect to the ion’s position; hence approximating the energy profile as a linear change will make it easier to obtain the mathematical relationship, as it will be explained in the following paragraphs.

Accordingly, the barrier energy of the closed hydrophobic gate can be described by the following equation according to the green curve in Figure 1:

(2) $$U(x)=\frac{g}{w}x$$

where *g* is the height of the triangle or the height of the energy barrier or simply ‘the energy of the gate’, *w* is the width of the barrier or the length of the gate, and *x* is the position of ion while passing through the closed gate.

The original equation that calculates the tunneling probability of a particle through a barrier of arbitrary shape [24]:

(3) $$T_{Q}=e^{\frac{-\sqrt{8m}}{\hbar }\int_{X1}^{X2}\sqrt{U(x)-KE}dx}$$

The integral in Equation (3) can be solved as the following:

(4) $$\int_{X1}^{X2}\sqrt{\frac{g}{w}x-KE}dx=\frac{2w}{3g}\sqrt{{\left(\frac{g}{w}x_{2}-KE\right)}^{3}}-\frac{2w}{3g}\sqrt{{\left(\frac{g}{w}x_{1}-KE\right)}^{3}}$$

*x*_2_ is at the end of the gate ($x_{2}=w$) and *x*_1_ is where *U*(*x*_1_) = *KE*, hence Equation (4) becomes:

(5) $$\int_{X1}^{X2}\sqrt{\frac{g}{w}x-KE}dx=\frac{2w}{3g}\sqrt{{\left(g-KE\right)}^{3}}$$

By substituting Equation (5) in Equation (3), we get Equation (1).

Our solved integral is based on the green curve in Figure 1, but if the integral is solved according to the red curve, we will get the same result as the following:

The left red line can be represented by the following equation $U(x)=\frac{2g}{w}x$, while the right red line can be represented by the following equation $U(x)=\frac{-2g}{w}(x-w)$ and if the integral is taken through the forbidden region from *x*_1_′ to *x*_2_′:

(6) $$\int_{x_{1}{}^{\prime }}^{\frac{w}{2}}\sqrt{\frac{2g}{w}x-KE}dx+\int_{\frac{w}{2}}^{{x_{2}}^{\prime }}\sqrt{\frac{-2g}{w}(x-w)-KE}dx=(\frac{w}{3g}\sqrt{{\left(\frac{2g}{w}(\frac{w}{2})-KE\right)}^{3}}-\frac{w}{3g}\sqrt{{\left(\frac{2g}{w}(x_{1}{}^{\prime })-KE\right)}^{3}})+(\frac{-w}{3g}\sqrt{{\left(\frac{-2g}{w}(x_{2}{}^{\prime }-w)-KE\right)}^{3}}-\frac{-w}{3g}\sqrt{{\left(\frac{-2g}{w}(\frac{w}{2}-w)-KE\right)}^{3}})$$

*x*_1_′ and *x*_2_′ are where *U*(*x*) = *KE*, hence:

(7) $$\int_{x_{1}{}^{\prime }}^{\frac{w}{2}}\sqrt{\frac{2g}{w}x-KE}dx+\int_{\frac{w}{2}}^{{x_{2}}^{\prime }}\sqrt{\frac{-2g}{w}(x-w)-KE}dx=\frac{w}{3g}\sqrt{{\left(g-KE\right)}^{3}}+\frac{w}{3g}\sqrt{{\left(g-KE\right)}^{3}}=\frac{2w}{3g}\sqrt{{\left(g-KE\right)}^{3}}$$

Accordingly, both barrier shapes are equivalent to estimate the tunneling probability because the solved integrals in both cases are the same. For this reason, we can say that the energy barrier of the hydrophobic gate can be viewed as the barrier shape of a regular electric field based on the approximations made on the actual curves of the energy profiles of ions while passing through the closed hydrophobic gate and hydrophobic membranes [17,18,19,20,21,22,23]. So, in a conclusion, the barrier shapes in Figure 1 are consistent and reasonable for the following reasons: (1) the curves in Figure 1 are triangle barriers similar to the barrier shapes obtained based on the potential mean force calculations in these references [17,18,19,20,21,22,23], (2) up to authors’ knowledge, there is no clear mathematical equation that predicts how the barrier energy changes with respect to the ion’s position in a hydrophobic material.

**Figure 2:**
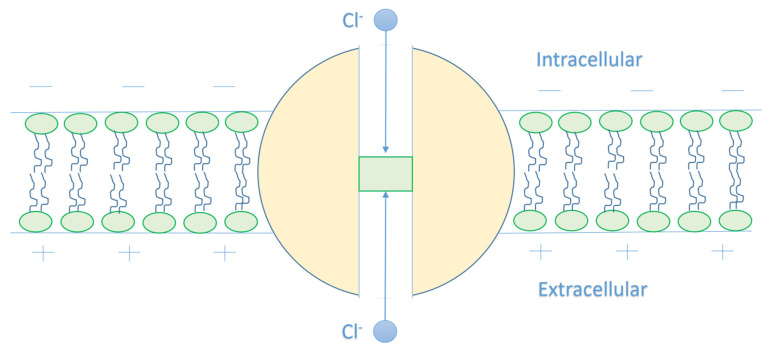
The figure represents a schematic diagram of chloride ion movement in the GABA receptor. The intracellular chloride ion moves from the negative side of the membrane towards the positive side, and this increases its kinetic energy until hitting the closed gate in the center of the GABA receptor, while the extracellular chloride ion moves from the positive side towards the negative side and this decreases its kinetic energy until hitting the closed gate.

As chloride ions are present in two compartments, which are intracellular and extracellular compartments, we expect different kinetic energy for the intracellular and extracellular chloride ions. As chloride ions are anions and the membrane potential of cells are negative inside with respect to outside, we expect that intracellular chloride ions will get a kinetic energy from the membrane potential as they go from the intracellular compartment to the extracellular compartment through the GABA receptor. However, the kinetic energy of the extracellular chloride ions will decrease as they go through the membrane potential. See Figure 2.

**Figure 3:**
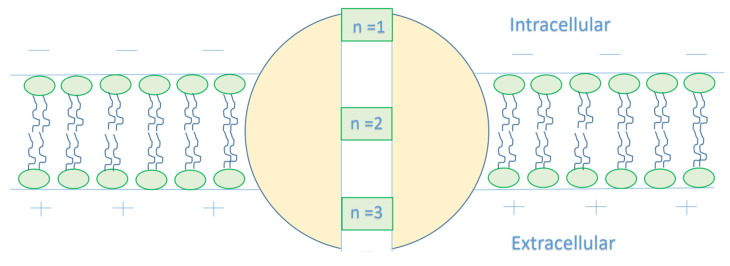
The figure represents different locations of the closed gate in the GABA receptor. The gate location affects the kinetic energy of the chloride ion once it reaches the closed gate. As the *n* value increases, the membrane potential available to enhance the kinetic energy of the intracellular anion increases, hence its kinetic energy increases. On the other hand, as the *n* value increases, the membrane potential available to dampen the kinetic energy of the extracellular anion decreases, hence the decrease in its kinetic energy is reduced.

As this article [15] indicates that the closed gate is in the middle of the GABA receptor, other locations cannot be excluded. Therefore, we will make our investigations based on different locations. We will refer to the location of the gate by *n* and we will choose three values for *n*, which are *n* = 1, *n* = 2, and *n* = 3, see Figure 3. Accordingly, this means that the kinetic energy of the extracellular chloride ion will decrease by $\frac{qV_{m}}{n}$, while the kinetic energy of the intracellular chloride ion will increase by $(1-\frac{1}{n})qV_{m}$.

Therefore, the kinetic energy of the intracellular chloride ions can be calculated by the following equation:

(8) $$KE\left(Cl_{I}\right)=KE_{Thermal}+qV_{m}(1-\frac{1}{n})$$

In addition, the kinetic energy of the extracellular chloride ions can be calculated by the following equation:

(9) $$KE(Cl_{E})=KE_{Thermal}-\frac{qV_{m}}{n}$$

where $KE_{Thermal}$ is the average thermal kinetic energy, *q* is the charge of chloride ion (1.6 × 10^−19^ C), *V_m_* is the cellular membrane potential, and *n* is the location of the gate.

If we take the average thermal energy $KE_{Thermal}=\frac{3}{2}K_{B}T=0.64\times 10^{-20}$ J for extracellular ions, this will result in a negative kinetic energy. For example, if *n* = 1 and *V_m_* = 0.07 V, then $KE(Cl_{E})=-0.48\times 10^{-20}$ J. Such a negative value can be used and substituted in Equation (1) to calculate the tunneling probability. However, to get a reasonable positive value of the kinetic energy of the extracellular ions, the following equation can be used:

(10) $$KE(Cl_{E})=\frac{0+(3K_{B}T-\frac{qV_{m}}{n})}{2}=\frac{3}{2}K_{B}T-\frac{qV_{m}}{2n}$$

Equation (10) gives the average kinetic energy of the extracellular chloride ions of the two values, which are 0 J and $2KE_{Thermal}-\frac{qV_{m}}{n}=2(\frac{3}{2}K_{B}T)-\frac{qV_{m}}{n}=3K_{B}T-\frac{qV_{m}}{n}$. The first value 0 J means that the membrane potential decreased the thermal energy of the chloride ion to zero once it reached the closed gate, while we choose $2KE_{Thermal}$ as an estimation of the highest kinetic energy possible with a reasonable fraction of chloride ions since increasing the thermal kinetic energy further will make the fraction of chloride ions much less to be reasonable to calculate the average kinetic energy. As we said, this approach is just to provide a more meaningful physical values of the kinetic energy and not to provide the exact values. 

Accordingly, the kinetic energy of the intracellular and the extracellular chloride ions can be calculated by the following equations, respectively:

(11) $$KE\left(Cl_{I}\right)=\frac{3}{2}K_{B}T+qV_{m}(1-\frac{1}{n})$$

(12) $$KE(Cl_{E})=\frac{3}{2}K_{B}T-\frac{qV_{m}}{2n}$$

where *K_B_* = 1.38 × 10^−23^ J/K and *T* is the body temperature (310 K).

Equation (12) calculates the average kinetic energy of the extracellular chloride ions that can reach the closed gate. In such way, if *n* = 1 and *V_m_* = 0.07 V, $KE(Cl_{E})=\frac{3}{2}K_{B}T-\frac{qV_{m}}{2n}=0.08\times 10^{-20}$ J. This value of kinetic energy is reasonable because it is a positive value and it will be less than the energy of the intracellular chloride ions if the same variables are substituted in Equation (11), which is $KE\left(Cl_{I}\right)=0.64\times 10^{-20}$ J. Moreover, if *V_m_* = 0 V, both intracellular and extracellular chloride ions have the same average kinetic energy $\frac{3}{2}K_{B}T$. In conclusion, Equations (11) and (12) are simple, but they are consistent and reasonable because they describe how the membrane potential changes the kinetic energy of the intracellular and extracellular chloride ions with meaningful physical values, also in the absence of membrane potential, both of them have the same average kinetic energy. This is said because choosing values higher or less than 2*KE_Thermal_* will result in a discrepancy between the intracellular and extracellular chloride ions when the case is *V_m_* = 0 V, which is not consistent because both of them should have the same average thermal kinetic energy in such a case.

Accordingly, the quantum tunneling probability of the intracellular chloride ions can be calculated by the following equation:

(13) $$T_{Q}(Cl_{I})=e^{\frac{-\sqrt{8m}}{\hbar }\times \frac{2w}{3g}\sqrt{{\left(g-(qV_{m}(1-\frac{1}{n})+\frac{3}{2}K_{B}T)\right)}^{3}}}$$

while the quantum tunneling probability of the extracellular chloride ions can be calculated by the following equation:

(14) $$T_{Q}(Cl_{E})=e^{\frac{-\sqrt{8m}}{\hbar }\times \frac{2w}{3g}\sqrt{{\left(g-(\frac{3}{2}K_{B}T-\frac{qV_{m}}{2n})\right)}^{3}}}$$

To simplify Equations (13) and (14), the following mathematical simplifications will be considered: we take 10^−20^ as a common factor from the square root and multiply the exponent by $\sqrt{{\left(10^{-20}\right)}^{3}}=10^{-30}$$\sqrt{{\left(10^{-20}\right)}^{3}}=10^{-30}$, we take $L=\frac{w}{10^{-10}}$ and multiply the exponent by 10^−10^ and we take $G=\frac{g}{10^{-20}}$ and divide the exponent by 10^−20^. Therefore, when we say for example that the energy of the gate *G* = 2 J, then the actual energy of the gate *g* = 2 × 10^−20^ J. Also, when we say that L = 1 m, then the actual gate length *w* = 1 × 10^−10^ m. Eventually, Equations (13) and (14) become:

(15) $$T_{Q}(Cl_{I})=e^{-M(Cl)\times \frac{L}{G}\sqrt{{\left(G-16V_{m}(1-\frac{1}{n})-0.64\right)}^{3}}}$$

(16) $$T_{Q}(Cl_{E})=e^{-M(Cl)\times \frac{L}{G}\sqrt{{\left(G-0.64+\frac{8V_{m}}{n}\right)}^{3}}}$$

where *M*(*Cl*) is just a factor that contains the mass of the ion, the reduced Planck constant and the multiplying and dividing factors that are the result of the simplifications made above.

Hence, $M(Cl)=\frac{\sqrt{8m}}{\hbar }\times \frac{2\times 10^{-10}\times 10^{-30}}{3\times 10^{-20}}=\frac{\sqrt{8\times 5.9\times 10^{-26}}}{1.05\times 10^{-34}}\times \frac{2\times 10^{-20}}{3}=43.62$.

The quantum tunneling of ions can result in a quantum unitary conductance of the closed GABA receptors. To obtain the quantum unitary conductance of the closed GABA receptors, the following equation will be applied [24,25]:

(17) $$C_{Q-GABA}=\frac{q^{2}}{h}T_{Q}\left(Cl\right)$$

where *q* is the charge of chloride ion (1.6 × 10^−19^ C), *h* is the Planck constant (6.6 × 10^−34^ Js), and *T_Q_*(*Cl*) is the quantum tunneling probability of chloride ions.

Eventually, the quantum membrane conductance at certain density *D* of GABA receptors can be calculated by the following equation [26]:

(18) $$MC_{Q-GABA}=D\times C_{Q-GABA}$$

To assess the influence of quantum membrane conductance induced by the quantum tunneling of chloride ions on the resting membrane conductance, the Goldman–Hodgkin–Katz (GHK) equation should be used. Before going through the effect of the quantum tunneling of chloride ions, we should first determine the membrane potential by sodium and potassium ions at the physiological parameters. To achieve this, the classical version of the GHK should be used [26]:

(19) $${\left[K\right]}_{E}MC_{K}+{\left[Na\right]}_{E}MC_{Na}=e^{\frac{-FV_{m}}{RT}}({\left[K\right]}_{I}MC_{K}+{\left[Na\right]}_{I}MC_{Na})$$

where [*K*]*_E_* is the extracellular potassium concentration, [*K*]*_I_* is the intracellular potassium concentration, [*Na*]*_E_* is the extracellular sodium concentration, [*Na*]*_I_* is the intracellular sodium concentration, *MC_K_* is the leaky membrane conductance of potassium ions at the resting, *MC_Na_* is the leaky membrane conductance of sodium ions at the resting state, *F* is Faraday’s constant (96,485.33 C/mol), *R* is the gas constant (8.31 J/Kmol), *T* is the body temperature (310 K), and *V_m_* is the membrane potential.

We added the minus sign in this term $e^{\frac{-FV_{m}}{RT}}$ to get an absolute value of the membrane potential, which is negative inside with regard to outside. Hence, we will deal with positive values of membrane potential in the following sections but it is considered to be negative inside with regard to outside.

In our paper, we set the following values: [*K*]*_E_* = 4 mmol/L [26], [*Na*]*_E_* = 142 mmol/L [26], [*K*]*_I_* = 140 mmol/L [26], [*Na*]*_I_* = 14 mmol/L [26], *MC_K_* = 0.5 mS/cm^2^ [26], and *MC_Na_* = 0.022 mS/cm^2^ [26] in Equation (19) to get a membrane potential of 0.07 V. These values will be the reference values of investigation in the present study to show how the quantum tunneling of chloride ions can change the membrane potential value.

To assess the effect of the classical transport of chloride ions through the open GABA receptors, the following equation can be used [26]:

(20) $${\left[K\right]}_{E}MC_{K}+{\left[Na\right]}_{E}MC_{Na}+{\left[Cl\right]}_{I}MC_{C-GABA}=e^{\frac{-FV_{m}}{RT}}({\left[K\right]}_{I}MC_{K}+{\left[Na\right]}_{I}MC_{Na}+{\left[Cl\right]}_{E}MC_{C-GABA})$$

where [*Cl*]*_I_* is the intracellular chloride concentration, [*Cl*]*_E_* is the extracellular chloride concentration, and *MC_C−GABA_* is the membrane conductance of chloride ions due to the classical opening of the GABA receptor.

On the other hand, to show how the quantum tunneling of chloride ions through the closed GABA receptors affect the resting membrane potential, the quantum version of the GHK equation is used [27]:

(21) $${\left[K\right]}_{E}MC_{K}+{\left[Na\right]}_{E}MC_{Na}+{\left[Cl\right]}_{I}MC_{Q-GABA}(I)=e^{\frac{-FV_{m}}{RT}}({\left[K\right]}_{I}MC_{K}+{\left[Na\right]}_{I}MC_{Na}+{\left[Cl\right]}_{E}MC_{Q-GABA}(E))$$

where $MC_{Q-GABA}(I)$ is the quantum membrane conductance of the intracellular chloride ions and $MC_{Q-GABA}(E)$ is the quantum membrane conductance of the extracellular chloride ions.

The values of chloride concentration are [*Cl*]*_E_* = 120 mmol/L [26] and [*Cl*]*_I_* = 5 mmol/L [26].

## 3. Results

In this section, we will evaluate the values of quantum tunneling probability of chloride ions through the closed GABA receptors, the values of quantum conductance of GABA receptors, and the values of quantum membrane conductance of GABA receptors. Eventually, we will be able to investigate the effect of quantum tunneling of chloride ions on the resting membrane potential.

The work that GABA agonist performs to activate the GABA receptors is called the energy of activation. The energy of activation is a reasonable estimation for the energy barrier height of the closed gate of GABA receptors because agonists such as GABA induce conformational changes to open the closed gate, hence what prevents chloride ion from passing this closed gate is the energy required to open it. The activation energy required to open the closed GABA receptors by GABA ligand is 4.3 kcal/mol = 3 × 10^−20^ J [28]. However, a more accurate estimation of the barrier height should be made based on the calculations of potential mean force (PMF) while ion passes through the closed gate. Up to authors’ knowledge, there is no evidence on the exact value of the barrier height for the closed gate of GABA receptor, but as GABA receptors are similar to the other pentameric ligand-gated ion channels such as nACh receptors and 5-HT3 receptors, then the obtained value of 10*K_B_T* = 4.3 × 10^−20^ J from the closed nACh receptors can be used to estimate the barrier height of the closed gate for GABA receptor [17,18]. In both ways of estimation, the energy barrier is within the order of magnitude 10^−20^. Accordingly, we are going to choose the range (2 − 6) × 10^−20^ J for the barrier height *g* with an average value 4 × 10^−20^ J to evaluate its influence on the quantum tunneling of chloride ions. The most important aspect of this range is that its values are within the order of magnitude of 10^−20^, which is consistent with the experimental observations. Moreover, we will show later that the influential quantum tunneling, which affects the membrane potential, occurs at lower values and around 2 × 10^−20^ J because the quantum model explains the depolarization effect of GABA receptors based on the drop in the height of the energy barrier possibly due to either physiological or pathological conditions. Hence, any value higher than 2 × 10^−20^ J will not be significant to enhance the quantum tunneling of chloride ions. This indicates that the energy barrier made by the hydrophobic gate is high enough to block the classical permeation and the quantum tunneling at normal conditions in which the barrier is higher than 2 × 10^−20^ J.

Additionally, the closed gate is formed by the aggregation of amino-acid residues. Hence, it is expected that the length of the gate equals the length of a residue within the alpha-helix 1.5 × 10^−10^ m [29,30]. In our study, the length range (1 − 2) × 10^−10^ m is considered for the purposes of evaluation with an average value of 1.5 × 10^−10^ m. Moreover, we will show how the length of the gate can influence the quantum tunneling-induced membrane depolarization by setting values higher than 1.5 × 10^−10^ m in case each subunit of GABA receptor contributes by more than one residue to the hydrophobic gate.

The range of gate location *n* (1–3) is considered for the purposes of evaluation with an average value of 2 (at the middle of the GABA receptor) to account for the different possible locations of the gate in the GABA receptor. Moreover, the membrane potential range 0–0.07 V is considered for the purposes of investigation.

### 3.1. The Quantum Tunneling Probability of Chloride Ions through the Closed GABA Receptors

The quantum tunneling probability of intracellular chloride ions and extracellular chloride ions can be calculated by the following equations, respectively:

(22) $$T_{Q}(Cl_{I})=e^{-43.62\times \frac{L}{G}\sqrt{{\left(G-16V_{m}(1-\frac{1}{n})-0.64\right)}^{3}}}$$

(23) $$T_{Q}(Cl_{E})=e^{-43.62\times \frac{L}{G}\sqrt{{\left(G-0.64+\frac{8V_{m}}{n}\right)}^{3}}}\;$$

**Figure 4:**
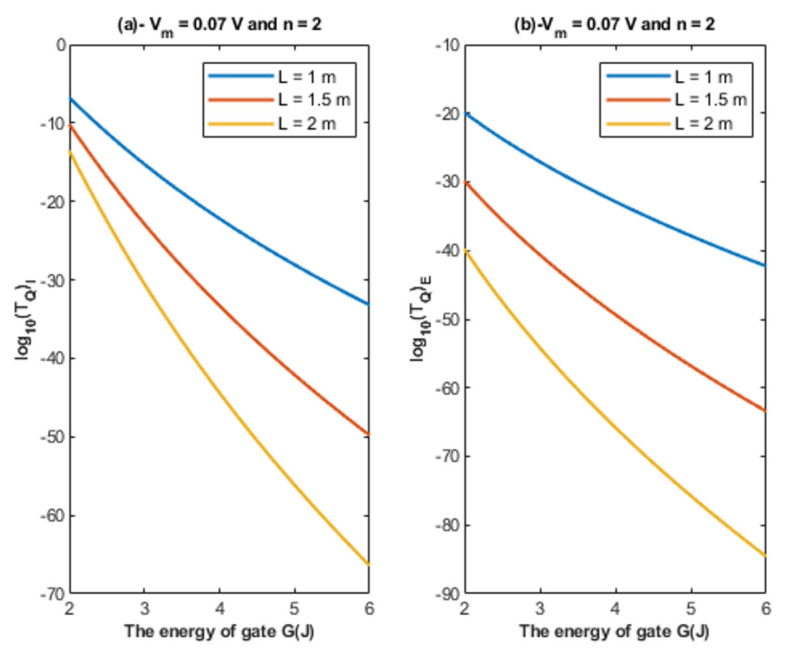
(**a**,**b**): The figure represents the relationship between the energy of the gate *G* and the common logarithm of the tunneling probability of the intracellular chloride ions $\log_{10}{\left(T_{Q}\right)}_{I}$ and the tunneling probability of the extracellular chloride ions $\log_{10}{\left(T_{Q}\right)}_{E}$, respectively. The relationships are investigated at different values of gate length and according to the setting values above each figure.

Based on Equations (22) and (23), the relationship between the energy of the gate and the tunneling probability of chloride ions is represented at different values of gate length to show its influence on this relationship. See Figure 4.

**Figure 5:**
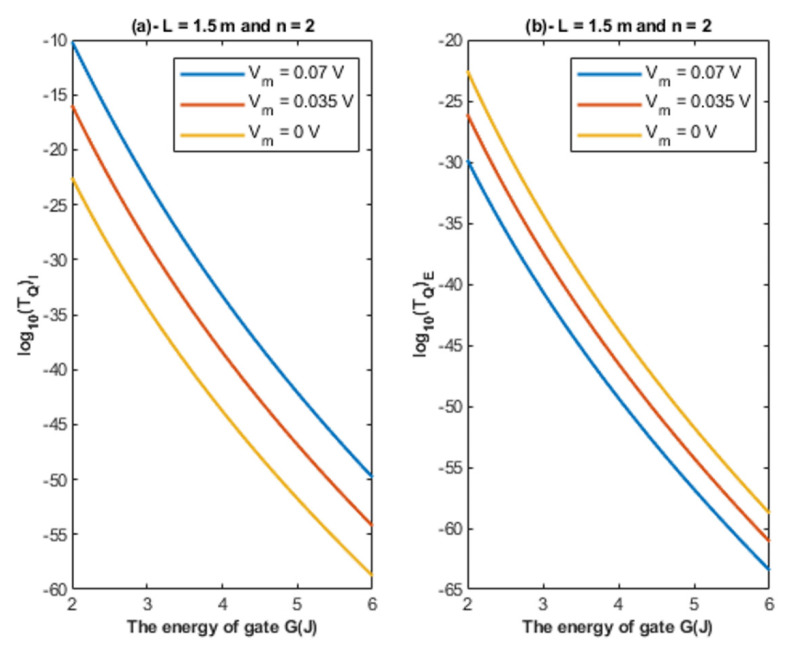
(**a**,**b**): The figure represents the relationship between the energy of gate *G* and the common logarithm of the tunneling probability of the intracellular chloride ions $\log_{10}{\left(T_{Q}\right)}_{I}$ and the tunneling probability of the extracellular chloride ions $\log_{10}{\left(T_{Q}\right)}_{E}$, respectively. The relationships are investigated at different values of membrane potential and according to the setting values indicated above each graph.

Based on Equations (22) and (23), the relationship between the energy of the gate and the tunneling probability of chloride ions is represented at different values of membrane potential to show its influence on this relationship. See Figure 5.

**Figure 6:**
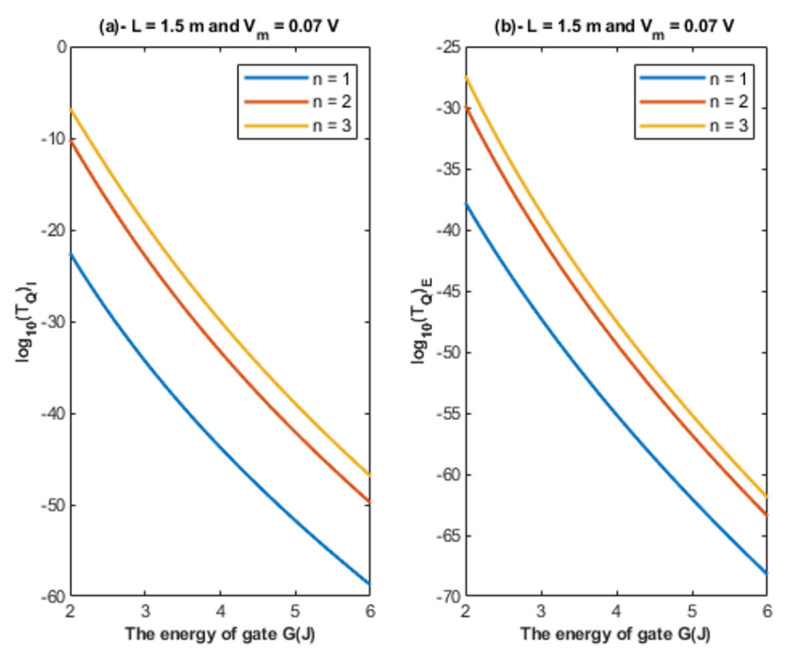
(**a**,**b**): The figure represents the relationship between the energy of gate *G* and the common logarithm of the tunneling probability of the intracellular chloride ions $\log_{10}{\left(T_{Q}\right)}_{I}$ and the tunneling probability of the extracellular chloride ions $\log_{10}{\left(T_{Q}\right)}_{E}$, respectively. The relationships are investigated at different values of gate location and according to the setting values indicated above each graph.

Based on Equations (22) and (23), the relationship between the energy of the gate and the tunneling probability of chloride ions is represented at different values of gate location to show its influence on this relationship. See Figure 6.

### 3.2. The Quantum Unitary Conductance of Closed GABA Receptors Mediated by the Quantum Tunneling of Chloride Ions

The quantum unitary conductance of closed GABA receptors for the intracellular chloride ions and the extracellular chloride ions can be calculated by the following equations, respectively:

(24) $$C_{Q-GABA}(I)=3.88\times 10^{-5}\times e^{-43.62\times \frac{L}{G}\sqrt{{\left(G-16V_{m}(1-\frac{1}{n})-0.64\right)}^{3}}}$$

(25) $$C_{Q-GABA}(E)=3.88\times 10^{-5}\times^{-43.62\times \frac{L}{G}\sqrt{{\left(G-0.64+\frac{8V_{m}}{n}\right)}^{3}}}$$

where $\frac{q^{2}}{h}=\frac{{\left(1.6\times 10^{-19}\right)}^{2}}{6.6\times 10^{-34}}=3.88\times 10^{-5}$ S. The unit of quantum unitary conductance used in the present study is Siemens (S).

**Figure 7:**
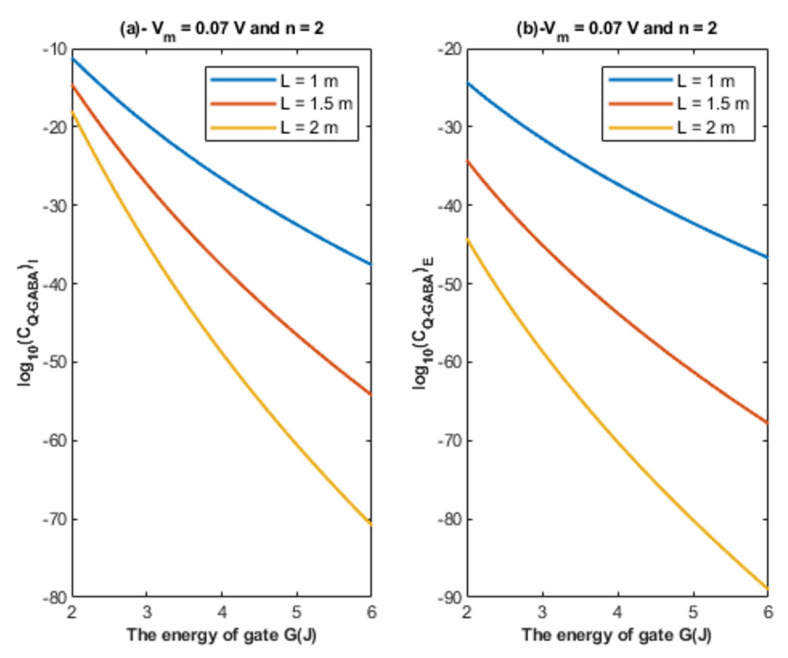
(**a**,**b**): The figure represents the relationship between the energy of the gate *G* and the common logarithm of the quantum unitary conductance of the intracellular chloride ions $\log_{10}{\left(C_{Q-GABA}\right)}_{I}$ and the quantum unitary conductance of the extracellular chloride ions $\log_{10}{\left(C_{Q-GABA}\right)}_{E}$, respectively. The relationships are investigated at different values of gate length and according to the setting values above each graph.

Based on Equations (24) and (25), the relationship between the energy of the gate and the quantum unitary conductance is represented at different values of gate length. See Figure 7.

**Figure 8:**
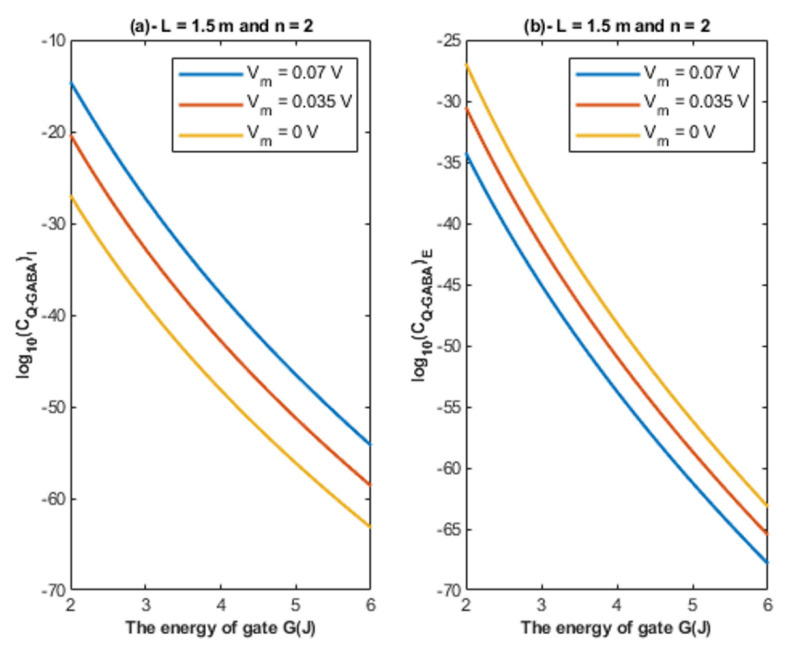
(**a**,**b**): The figure represents the relationship between the energy of the gate *G* and the common logarithm of the quantum unitary conductance of the intracellular chloride ions $\log_{10}{\left(C_{Q-GABA}\right)}_{I}$ and the quantum unitary conductance of the extracellular chloride ions $\log_{10}{\left(C_{Q-GABA}\right)}_{E}$, respectively. The relationships are investigated at different values of membrane potential and according to the setting values above each graph.

Based on Equations (24) and (25), the relationship between the energy of the gate and the quantum unitary conductance is represented at different values of membrane potential. See Figure 8.

**Figure 9:**
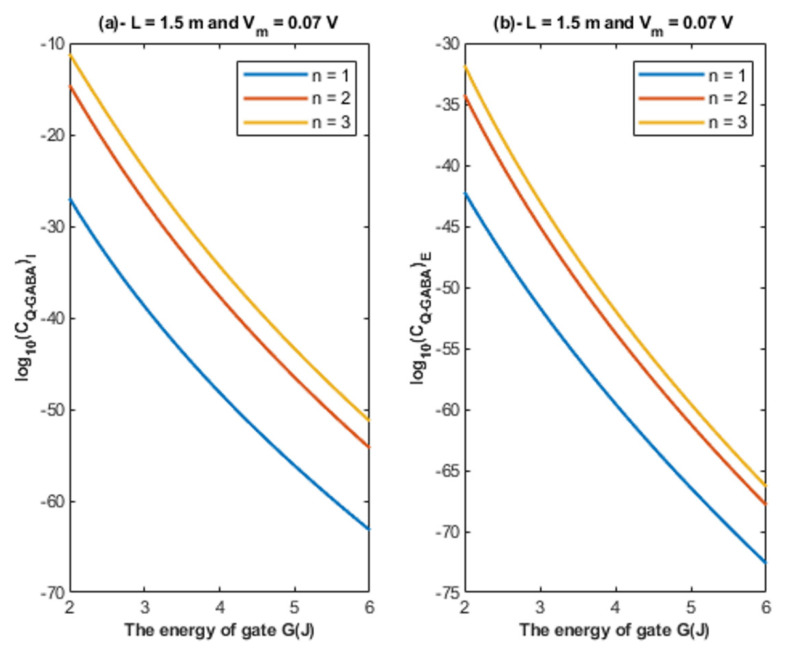
(**a**,**b**): The figure represents the relationship between the energy of the gate *G* and the common logarithm of the quantum unitary conductance of the intracellular chloride ions $\log_{10}{\left(C_{Q-GABA}\right)}_{I}$ and the quantum unitary conductance of the extracellular chloride ions $\log_{10}{\left(C_{Q-GABA}\right)}_{E}$, respectively. The relationships are investigated at different values of gate location and according to the setting values above each graph.

Based on Equations (24) and (25), the relationship between the energy of the gate and the quantum unitary conductance is represented at different values of gate location. See Figure 9.

### 3.3. The Quantum Membrane Conductance from the Closed GABA Receptors

The quantum membrane conductance from the closed GABA receptors for the intracellular chloride ions and the extracellular chloride ions can be calculated by the following equations, respectively:

(26) $$MC_{Q-GABA}(I)=3.88\times 10^{-2}\times e^{-43.62\times \frac{L}{G}\sqrt{{\left(G-16V_{m}(1-\frac{1}{n})-0.64\right)}^{3}}}\times D$$

(27) $$MC_{Q-GABA}(E)=3.88\times 10^{-2}\times^{-43.62\times \frac{L}{G}\sqrt{{\left(G-0.64+\frac{8V_{m}}{n}\right)}^{3}}}\times D$$

where 3.88 × 10^−5^ S is converted to 3.88 × 10^−2^ mS by multiplying by 10^3^ and *D* is the GABA receptors density (channels/cm^2^). Hence, the unit of the quantum membrane conductance used in the present study is mS/cm^2^. We will use three different values of GABA receptors density [31] to observe its influence on quantum conductance. These three values are: 10 receptors/µm^2^ (10^9^ channels/cm^2^), 100 receptors/µm^2^ (10^10^ channels/cm^2^), and 1000 receptors/ µm^2^ (10^11^ channels/cm^2^).

**Figure 10:**
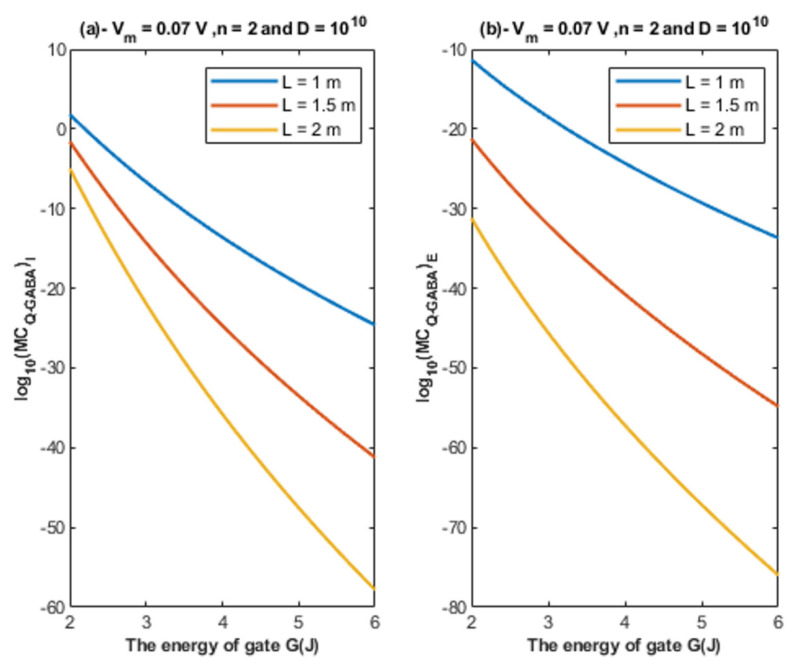
(**a**,**b**): The figure represents the relationship between the energy of the gate and the common logarithm of the quantum membrane conductance of the intracellular chloride ions $\log_{10}{\left(MC_{Q-GABA}\right)}_{I}$ and the quantum membrane conductance of the extracellular chloride ions $\log_{10}{\left(MC_{Q-GABA}\right)}_{E}$, respectively. The relationships are investigated at different values of gate length and according to the setting values above each graph.

Based on Equations (26) and (27), the relationship between the energy of the gate and the quantum membrane conductance is represented at different values of gate length. See Figure 10.

**Figure 11:**
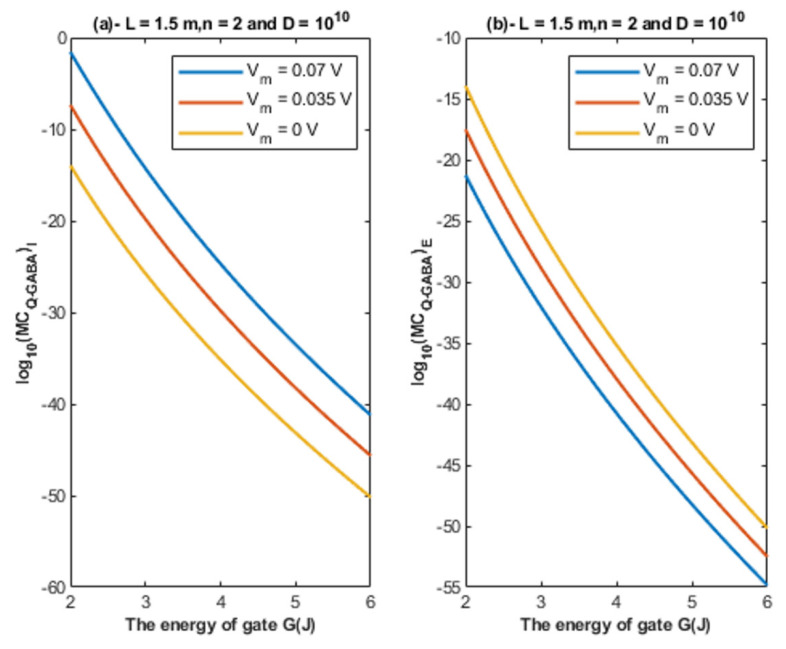
(**a,b**): The figure represents the relationship between the energy of the gate and the common logarithm of the quantum membrane conductance of the intracellular chloride ions $\log_{10}{\left(MC_{Q-GABA}\right)}_{I}$ and the quantum membrane conductance of the extracellular chloride ions $\log_{10}{\left(MC_{Q-GABA}\right)}_{E}$, respectively. The relationships are investigated at different values of membrane potential and according to the setting values above each graph.

Based on Equations (26) and (27), the relationship between the energy of the gate and the quantum membrane conductance is represented at different values of membrane potential. See Figure 11.

**Figure 12:**
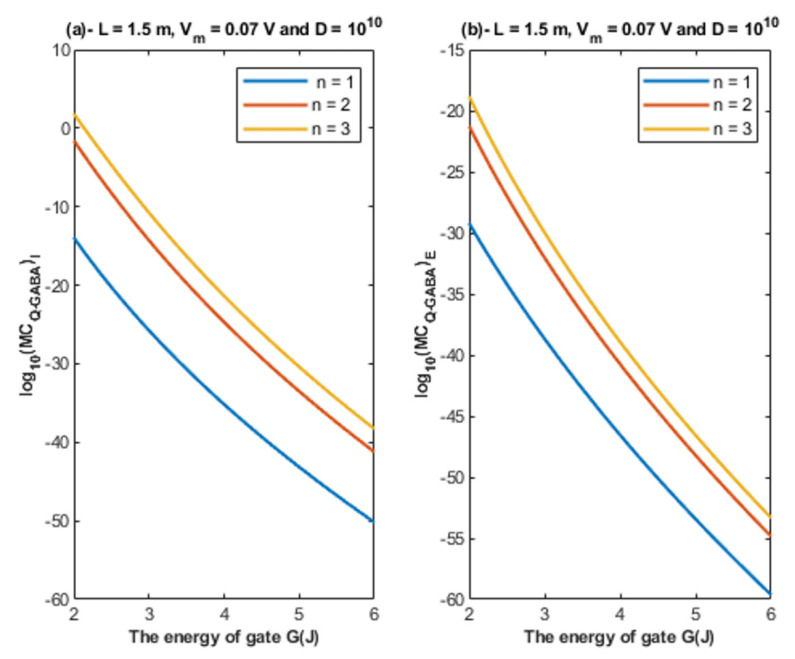
(**a,b**): The figure represents the relationship between the energy of the gate *G* and the common logarithm of the quantum membrane conductance of the intracellular chloride ions $\log_{10}{\left(MC_{Q-GABA}\right)}_{I}$ and the quantum membrane conductance of the extracellular chloride ions $\log_{10}{\left(MC_{Q-GABA}\right)}_{E}$, respectively. The relationships are investigated at different values of gate location and according to the setting values above each graph.

Based on Equations (26) and (27), the relationship between the energy of the gate and the quantum membrane conductance is represented at different values of gate location. See Figure 12.

**Figure 13:**
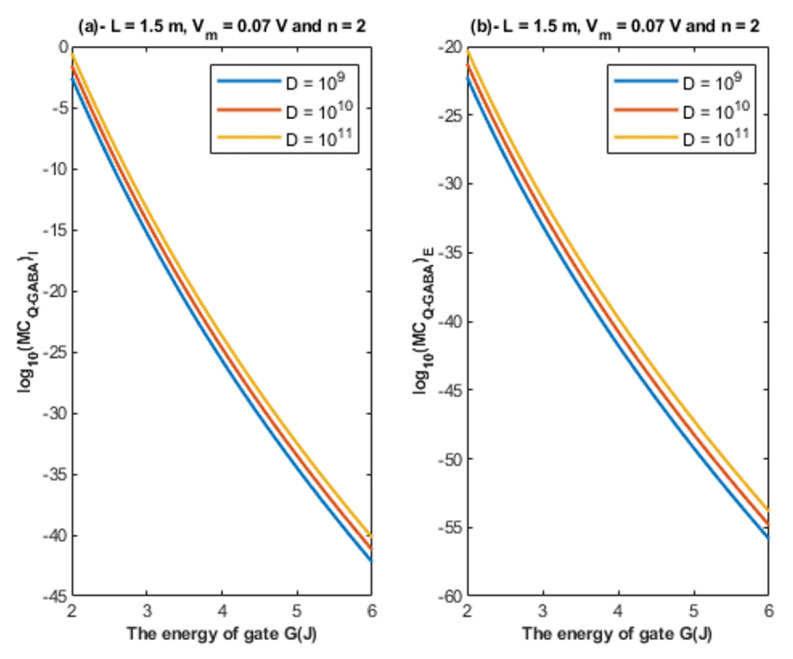
(**a**,**b**): The figure represents the relationship between the energy of gate *G* and the common logarithm of the quantum membrane conductance of the intracellular chloride ions $\log_{10}{\left(MC_{Q-GABA}\right)}_{I}$ and the quantum membrane conductance of the extracellular chloride ions $\log_{10}{\left(MC_{Q-GABA}\right)}_{E}$, respectively. The relationships are investigated at different values of receptors density and according to the setting values above each graph.

Based on Equations (26) and (27), the relationship between the energy of the gate and the quantum membrane conductance is represented at different values of receptors density. See Figure 13.

### 3.4. The Quantum Tunneling-Induced Membrane Depolarization via Quantum Tunneling of Chloride Ions through the Closed GABA Receptors

We substitute the values of ions concentration and their conductance values in Equation (21) to get the following equation:

(28) $$5.12+0.19\times D\times e^{-43.62\times \frac{L}{G}\sqrt{{\left(G-16V_{m}(1-\frac{1}{n})-0.64\right)}^{3}}}=e^{-37.45V_{m}}(70.31+4.66\times D\times e^{-43.62\times \frac{L}{G}\sqrt{{\left(G-0.64+\frac{8V_{m}}{n}\right)}^{3}}})$$

Equation (28) enables us to assess the influence of the quantum tunneling of chloride ions according to several factors including: the energy of the gate, the length of the gate, and the location of the gate.

**Figure 14:**
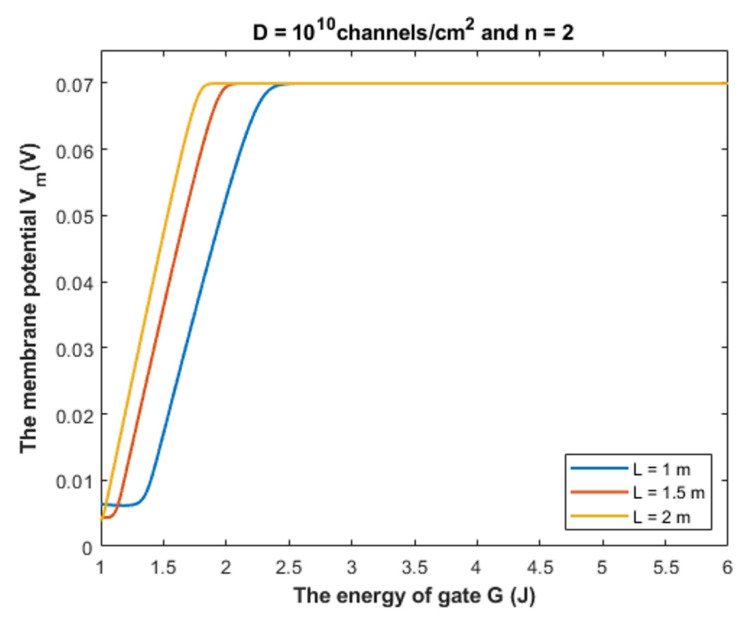
The figure represents the influence of the energy of gate *G* on the resting membrane potential according to different values of gate length and according to the setting values above the figure. The observed depolarization effect is due to quantum tunneling of chloride ions through GABA receptors.

Based on Equation (28), we can investigate the influence of gate energy on the resting membrane potential at different values of gate length. See Figure 14.

**Figure 15:**
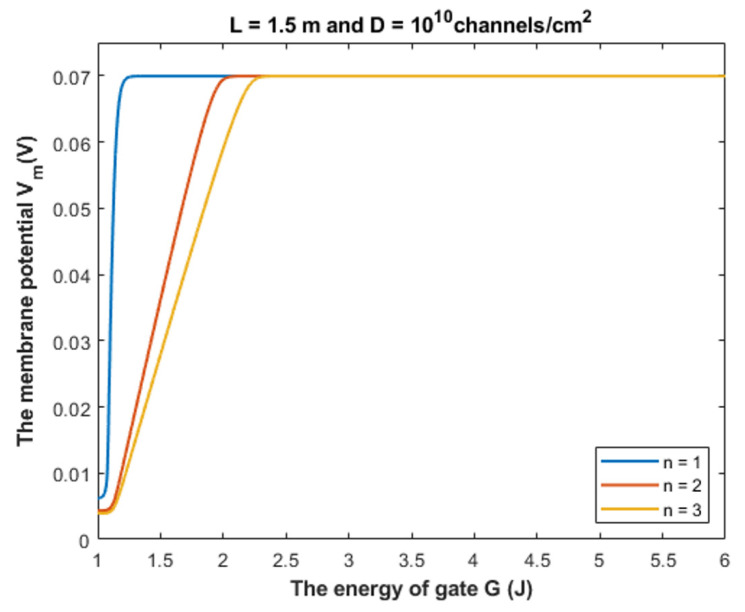
The figure represents the influence of the energy of gate *G* on the resting membrane potential according to different values of gate location and according to the setting values above the figure.

Based on Equation (28), we can investigate the influence of the gate energy on the resting membrane potential at different values of gate location. See Figure 15.

**Figure 16:**
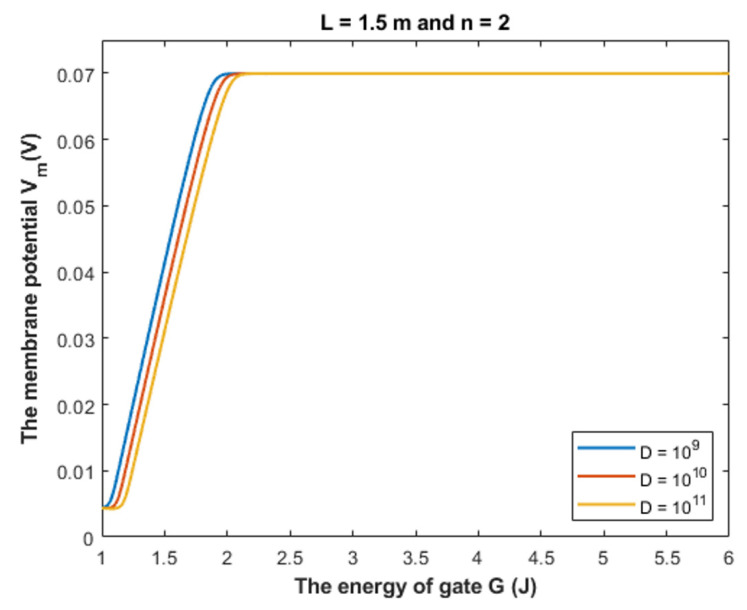
The figure represents the influence of the energy of gate *G* on the resting membrane potential according to different values of receptors density and according to the setting values above the figure.

Based on Equation (28), we can investigate the influence of the gate energy on the resting membrane potential at different values of receptors density. See Figure 16. 

### 3.5. The Effect of the Intracellular Chloride Concentration on the Resting Membrane Potential Based on the Quantum and Classical Models

**Figure 17:**
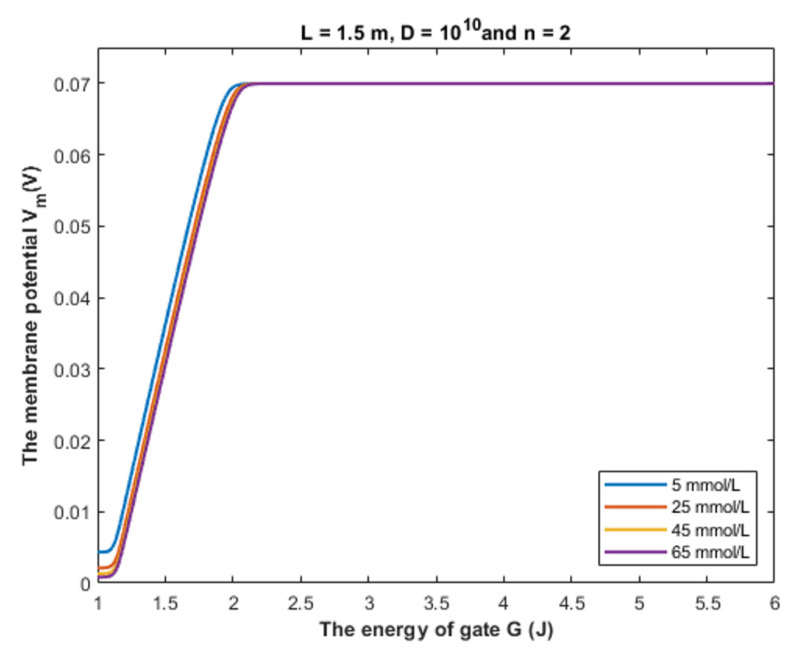
The figure represents the relationship between the energy of gate *G* and the resting membrane potential under the influence of the quantum tunneling of chloride ions at different intracellular chloride concentrations.

To investigate the influence of the intracellular chloride concentration in the context of the quantum tunneling of chloride ions, see Figure 17.

The graphs in Figure 17 are plotted according to Equation (28) by setting different values of intracellular chloride concentration. 

To investigate the influence of the intracellular chloride concentration on the resting membrane potential via the classical opening of certain numbers of GABA receptors, the concentration of ions and their conductance values are substituted in Equation (20) to get the following equation:

(29) $$5.12+C_{C-GABA}\times D\times {\left[Cl\right]}_{I}=e^{-37.45V_{m}}(70.31+C_{C-GABA}\times D\times {\left[Cl\right]}_{E})$$

where *C_C−GABA_* is the classical unitary conductance of open GABA receptor (30 × 10^−12^ S [32]) and *D* is the density of GABA receptors. Moreover, $C_{C-GABA}\times D=MC_{C-GABA}$ is the equation of the membrane conductance due to the opening of GABA receptors.

**Figure 18:**
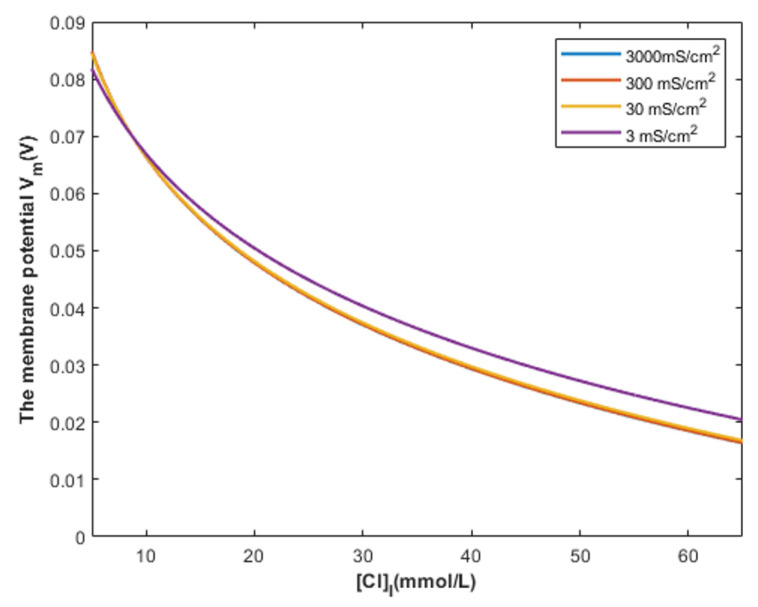
The figure represents the relationship between the intracellular chloride ions and the resting membrane potential under the influence of the classical transport of chloride ions through open GABA receptors at different values of membrane conductance of chloride ions.

According to Equation (29), the relationship between the intracellular chloride concentration and the resting membrane potential can be investigated at different values of membrane conductance from the opening of GABA receptors *MC_C−GABA_*. See Figure 18. 

## 4. Discussion

**Figure 19:**
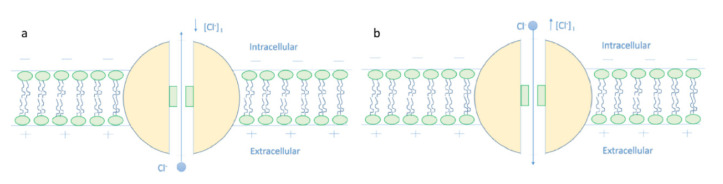
The figure represents the classical view that explains the depolarization action of GABA receptor. (**a**): The decrease in the intracellular chloride concentration will favor the influx of chloride ions that tends to hyperpolarize the membrane potential when the GABA receptor opens. (**b**): The increase in the concentration of the intracellular chloride ions will favor the efflux of chloride ions that tends to depolarize the resting membrane potential.

The present work introduces the mechanism of membrane depolarization by GABA receptors based on the quantum tunneling model. The basic idea behind this mechanism is that chloride ions can pass through the closed gate of GABA receptors via quantum tunneling. Quantum tunneling is a probabilistic quantum event; hence, the quantum tunneling probability should be calculated. To assess the influence of chloride ions quantum tunneling on the resting membrane conductance, the unitary quantum conductance of the GABA receptor and the quantum membrane conductance should be calculated. The following sections will focus on the numerical descriptions of these quantum electrical properties of GABA receptors and chloride ions to provide a better understanding of the depolarization action. The quantum tunneling model is proposed because it can explain the depolarization action of GABA receptors without the requirement of the intracellular chloride ions accumulation as in the classical interpretation, see Figure 19.

The increase in the intracellular chloride concentration may be overestimated because the measurement is based on the expression levels of chloride transporters and the experimental studies did not take into consideration the factor of neuronal activity, which affects the intracellular chloride concentration [8]. Therefore, the quantum model seems helpful to explain the depolarization effect in cases where the intracellular chloride concentration is normal.

**Figure 20:**
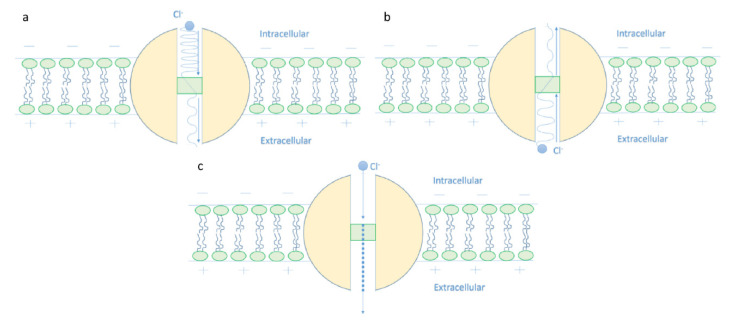
The figure represents the quantum mechanical view of the depolarization action of GABA receptor. (**a**): The intracellular chloride ion with higher kinetic energy, which is represented by a higher quantum wave frequency, has a higher quantum tunneling probability through the closed gate, which is represented by a higher wave amplitude after passing the gate. (**b**): The extracellular chloride ion with a lower kinetic energy, which is represented by a lower quantum wave frequency, has a lower quantum tunneling probability through the closed gate, which is represented by a lower wave amplitude after passing the gate. (**c**): Due to the discrepancy between the intracellular and extracellular chloride ions in terms of the tunneling probability, a quantum gradient favors the efflux of chloride ions, which will depolarize the membrane potential. The quantum efflux gradient is represented by a dotted line.

Based on Figure 4, Figure 5 and Figure 6:

1. It is clear that the tunneling probability of intracellular chloride ions is higher than that of extracellular chloride ions. This is attributed to the difference in the kinetic energy when they reach the closed gate. The higher the kinetic energy, the higher the tunneling probability. As the membrane potential is negative inside with regard to outside, it is expected that the intracellular chloride ion acquires kinetic energy while passing in GABA receptor until hitting the closed gate, while the extracellular chloride ions will lose kinetic energy as they pass in GABA receptors until reaching the closed gate. See Figure 20.

The case here is the opposite if it is compared with the case of cations such as lithium, sodium, and potassium ions [9,13] because the extracellular cations will get kinetic energy higher than the intracellular cations. Interestingly, such discrepancy in terms of tunneling probability generates what is called ‘quantum gradient’ that favors the efflux of chloride ions, which results in membrane depolarization, as will be discussed later.

2. The tunneling probability of both intracellular and extracellular chloride ions decreases as the energy of the gate and the length of the gate increase. On the other hand, the membrane potential has a differential effect on chloride ions. As the membrane potential increases, the tunneling probability of intracellular chloride ions increases, whereas the tunneling probability of extracellular chloride ions decreases as the membrane potential increases. Additionally, as the value of *n* increases, the membrane potential available for intracellular chloride ions increases and thus their tunneling probability increases. However, as the value of *n* increases, the membrane potential opposing the movement of extracellular chloride ions and the loss of their kinetic energy decrease and thus their tunneling probability increases.

The binding of GABA ligand to its receptor is required to open the closed gate by decreasing its energy barrier [28]. When the gate is open, the chloride ion can pass classically. The quantum tunneling model implies that chloride ions can pass through the closed gate at different levels of gate energy and without binding of GABA to its receptor. The values of tunneling probability at different levels of gate energy were clearly presented in the previous figures according to different factors. To determine the significance of this quantum tunneling, it is vital to consider the quantum unitary conductance of GABA receptor and to be compared with the classical conductance when the receptor is open.

Based on Figure 7, Figure 8 and Figure 9:

1. According to the quantum tunneling model, the quantum unitary conductance of the intracellular chloride ions is higher than the quantum conductance of the extracellular chloride ions. On the other hand, the classical model assigns the same unitary conductance for both extracellular and intracellular chloride ions.
2. If we take the classical unitary conductance of open GABA receptor to be 30 × 10^−12^ S [32], it seems obvious that the quantum unitary conductance is extremely small until reaching near *G* = 2 J, at which the quantum unitary conductance becomes comparable to the classical value. Therefore, it is expected that the quantum tunneling of chloride ions becomes influential when the energy of the gate of GABA receptors decreases to 2 J or less, as will be explained later in the paper. Based on the quantum tunneling model, it is predicted that GABA receptors have persistent basal activity in the absence of GABA ligands under certain conditions that can be physiological or pathological if they cause a decrease in the energy of the closed gate. The quantum tunneling effect will be extremely low at gate energy values higher than 2 J.
3. The quantum unitary conductance changes according to the energy of the gate, the length of the gate, the membrane potential, and the location of the gate as it was discussed regarding the tunneling probability.

Accordingly, the quantum membrane conductance due to quantum tunneling of chloride ions through the closed GABA receptors can be investigated. Thus, based on Figure 10, Figure 11, Figure 12 and Figure 13:

1. The quantum membrane conductance of intracellular chloride ions is higher than the quantum membrane conductance of the extracellular chloride ions. This discrepancy in the quantum conductance will result in a net efflux of chloride ions.
2. If the quantum conductance values are compared with the leaky classical conductance values of potassium and sodium ions at the resting state, which are 0.5 mS/cm^2^ and 0.022 mS/cm^2^, then it is clear that the values of quantum conductance for the intracellular ions become comparable to the classical ones once the energy of the closed gate drops to 2 J. This is said to provide an empirical prediction regarding the influence of quantum tunneling on the resting membrane potential. As the quantum conductance of the extracellular ions remain extremely low across all different values of gate energy and the quantum conductance of the intracellular ions becomes comparable to the leaky conductance of sodium and potassium ions around *G* = 2 J, then it is expected that chloride ions can depolarize the resting membrane potential via quantum tunneling through the closed GABA receptors.

### 4.1. The Influence of Chloride Ions Quantum Tunneling on the Resting Membrane Potential

#### 4.1.1. The Influence of Quantum Tunneling of Chloride Ions at Different Values of Gate Length L

Equation (28) is used to investigate the influence of chloride ions quantum tunneling on the resting membrane potential. According to the quantum tunneling model, it is expected that chloride ions will depolarize the resting membrane potential because intracellular chloride ions have higher quantum membrane conductance if they are compared with the extracellular ions. This will result in a net chloride efflux that depolarizes the membrane potential. To make the depolarization effect of GABA receptors more understandable, a numerical description will be useful. 

Based on Figure 14, Figure 15 and Figure 16, it is clear that the membrane potential remains unaffected across a certain range of gate energy until reaching a gate energy value called the energy curving point *G_cur_* at which the action of depolarization begins. To calculate this value, we choose *V_m_* = 0.086 V to be the beginning value of depolarization then we substitute it in Equation (28) and solve the equation for *G* to get *G_cur_* based on the setting values in Figure 14, Figure 15 and Figure 16. By looking at the mathematical graphs, it is clear that the depolarization starts near the value of *G* = 2 J. The end of the graphs is at *G* = 1 J. Hence, the rate of depolarization can be calculated by the following equation:

(30) $$R=\frac{0.086-V_{m}(G=1)}{G_{cur}-1}\;$$

We used the value *G* = 1 J and we did not use it before in the discussion of tunneling probability and quantum conductance to ensure that the energy barrier of the closed gate *G* is higher than the kinetic energy of the ion so that the principle of quantum tunneling can be applied; otherwise, the ion can pass classically through the gate. Moreover, Equation (28) is more dynamical equation if it is compared with the equations of tunneling probability and equations of quantum conductance because it take into consideration the interactions of two factors, which are the membrane potential and the energy of the gate, hence longer range of gate energy can be used.

To provide a numerical description of the depolarization action of GABA receptors at different values of gate length, see Table 1.

As the value of $G_{cur}$ increases, this means that the GABA receptor is more sensitive to the drop in the energy of the gate to depolarize the resting membrane potential because it will be more able to depolarize the membrane potential at higher values of *G*. 

Based on Table 1, we can conclude that as the gate length increases, the closed GABA receptor becomes less sensitive to depolarize the membrane potential. This is indicated by the decreasing values of *G_cur_*. On the other hand, the rate of depolarization increases as the gate length increases to compensate for the decreasing sensitivity.

**Figure 21:**
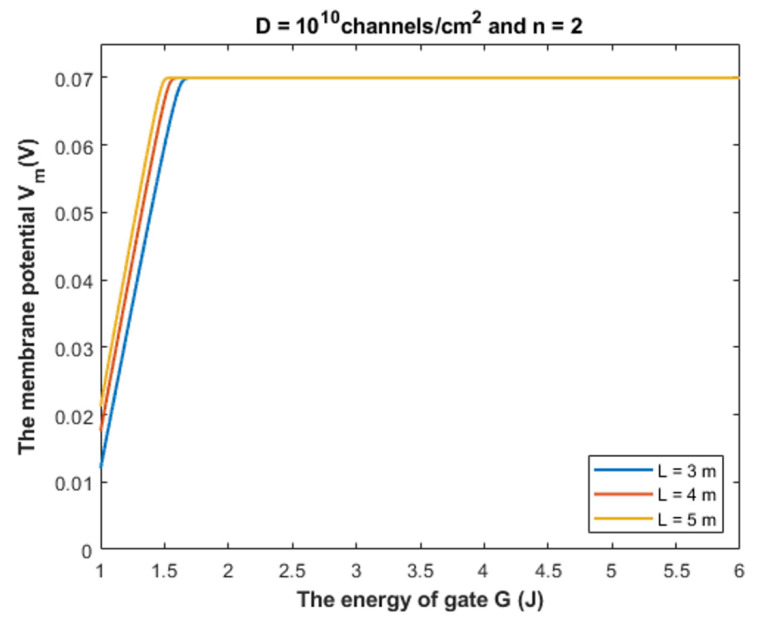
The figure represents the relationship between the energy of gate *G* and the membrane potential at gate length *L* values higher than 2 m to show its influence on the depolarization effect.

Our mathematical investigation in the present paper focuses on the range (1 − 2) × 10^−10^ m for the gate length. However, it will be useful to challenge the quantum model at higher values of the gate length. See Figure 21.

Figure 21 shows that the quantum tunneling-induced membrane depolarization is still evident and valid even at higher values of gate length in case there is more than one hydrophobic residue from each subunit of the GABA receptor that contributes to the hydrophobic gating. As the gate length *L* increases, the energy curving point decreases and the absolute value of membrane potential at *G* = 1 J increases (less depolarization). This means that as the gate length increases, the range of gate energy, in which the quantum tunneling can affect the membrane potential gets narrower. Herein, we stress on the idea that the kinetic energy of the ion is less than the energy of the gate across this range of influential quantum tunneling that can depolarize the membrane potential.

#### 4.1.2. The Influence of Quantum Tunneling of Chloride Ions at Different Values of Gate Location *n*

To provide a numerical description of the depolarization of GABA receptors at different values of gate location *n*, see Table 2.

Based on Table 2, we can conclude that as the value of gate location *n* increases, the closed GABA receptor becomes more sensitive to depolarize the resting membrane potential indicated by the increasing values of *G_cur_*. On the other hand, the rate of depolarization decreases as the *n* value increases.

#### 4.1.3. The Influence of Quantum Tunneling of Chloride Ions on the Resting Membrane Potential at Different Values of Channels Density D

To provide a numerical description of the depolarization by GABA receptors at different values of receptors density *D*, see Table 3.

Based on Table 3, we can conclude that as the receptors density increases, the closed GABA receptor becomes more sensitive to depolarize the resting membrane potential, which is indicated by the increasing values of *G_cur_*. On the other hand, the rate of depolarization decreases as the density of receptors increases. 

### 4.2. The Comparison between the Classical and Quantum Models of GABA Receptors in Terms of the Intracellular Chloride Concentration

According to Figure 17, it is obvious that the depolarization action mediated by the quantum tunneling of chloride ions is affected minimally by increasing the intracellular chloride concentration. Interestingly, even at intracellular chloride concentration of 5 mmol/L, closed GABA receptors can depolarize the resting membrane potential starting from *G* near 2 J until *G* = 1 J with a specific rate of depolarization. At the gate energy values higher than 2 J, the depolarization action via quantum tunneling is lost even at high intracellular chloride concentration because the tunneling probability will be extremely low to get significant quantum conductance to change the membrane potential, as we discussed before. 

On the other hand, according to Figure 18, the classical model predicts that depolarization action starts around the intracellular chloride concentration of 9 mmol/L. At intracellular concentrations less than 9 mmol/L, hyperpolarization is induced by GABA receptors opening because the absolute value of Nernst potential for chloride ions is higher than the absolute value of the initial membrane potential, which is 70 mV. Therefore, a net chloride influx occurs and manifests as hyperpolarization. 

Moreover, the quantum model is mainly concerned with the energy of the closed gate more than the chloride concentrations, while the classical model mainly depends on the intracellular concentration to explain the depolarization action. Therefore, the same intracellular chloride concentration can have different values of depolarized membrane potential according to the value of gate energy between 2 J and 1 J based on the quantum tunneling model. 

### 4.3. Possible Proposed Mechanisms behind the Drop in the Energy of the Closed Gate of GABA Receptors and Potential Implications and Perspectives

The major aim of the present article is to explain the depolarization action of GABA receptors according to the drop in the energy of the gate. This, as presented before, enables us to comprehend this effect under normal intracellular chloride concentration. In this section, we will propose certain mechanisms that can be responsible for the drop in the energy of the gate. These mechanisms may be used to provide more concrete approaches to test the quantum tunneling model of chloride ions.

The classical view of the GABA receptor function relies on the ability of the agonist, such as the GABA ligand, to open the closed gate by performing a work equal to the activation energy [28]. This gate is said to be open because its energy barrier is less than the kinetic energy of chloride ions, hence it allows the passage of both intracellular and extracellular chloride ions by the same permeability. The agonist decreases the energy barrier of the closed gate by dilating the pore and increasing the hydration probability [17,18]. Furthermore, the net flow is determined by the resting membrane potential and the Nernst potential of chloride ions, which is modulated by the intracellular chloride ions. On the other hand, the quantum tunneling model determines the function of GABA receptor according to the energy of the closed gate.

Accordingly, if certain physiological and pathological mechanisms decrease the energy of the closed gate, the quantum tunneling of chloride ions can be observed. However, the decrease in the energy of the gate must be made so that the gate is still closed and the energy barrier of the gate is higher than the kinetic energy of the chloride ion. According to the presented results, the critical energy value at which the depolarization effect starts is around 2 J. The major advantage of the quantum model over the classical model is that the explanation of the depolarization action of GABA receptors does not require the action of the intracellular accumulation of chloride ions. Therefore, the quantum model will be useful to explain the events of depolarization in which there is no chloride ions accumulation especially that the previously conducted studies might overestimate the values of the intracellular chloride concentration and did not take into consideration the neuronal activity that can influence the chloride concentrations. Thus, an alternative mechanism is required to explain such conditions of depolarization. 

Our aim by listing the following mechanisms is to show how the quantum tunneling model can be useful and how it can be exploited to be put under experimental investigation. Here we list a few possible mechanisms with potential implications:

1. We propose that during early development, the binding of GABA ligand to its receptor does not yield the full activation energy needed to open the closed gate; hence, the gate remains closed but with less energy barrier than before. Accordingly, the quantum tunneling of the intracellular chloride ions will be augmented more significantly than the tunneling of the extracellular chloride ions. This yields a quantum gradient that produces a net chloride efflux that leads to membrane depolarization, which has been observed during early neuronal development [2,3,4].
2. The gain-of-function mutations are well-established genetic conditions that affect ion channels such as ligand-gated channels and voltage-gated channels [33,34]. If these gain-of-function mutations decrease the gate energy of the closed GABA receptor, this will enhance the quantum tunneling of chloride ions to depolarize the membrane potential. Interestingly, this quantum tunneling-induced membrane depolarization occurs even without GABA binding to the receptor because the drop of the gate energy had occurred. Therefore, these mutations may be responsible for generating outward anion current that depolarizes membrane potential, which explains the increased tendency of seizures in patients with such mutations [33,34].
3. Acquired conditions such as stroke and trauma are implicated as pathological events that affect the function of GABA receptors [5,6]. As such conditions can decrease the energy required to open the voltage-gated channels [35,36], this can be extrapolated and applied on GABA receptors because they share the same hydrophobic gating mechanism [14,17]. Accordingly, these conditions can decrease the energy of the closed gate in GABA receptors, which leads to enhancing the quantum tunneling of chloride ions.
4. Since the depolarization mediated by GABA receptors can result in excitatory effects or inhibitory effects [8], this idea can be used to explain the mechanism of action of certain classes of agents and medications. These agents include the anesthetic agents that are used to shut down the consciousness so that certain surgery can be done. The classical view is that these agents activate the GABA receptors to induce hyperpolarizing currents and thus decrease neuronal activity [37,38]. In the perspective of the quantum tunneling model, these agents can hypothetically be viewed as quantum tunneling enhancers by lowering the values of gate energy facilitating the chloride efflux, which leads to quantum tunneling-induced membrane depolarization. This depolarization may inhibit neuronal activity, which explains the anesthetic effects of these agents. Moreover, the quantum tunneling model predicts the ability of chloride ions to depolarize the membrane potential by a large degree, reaching less than 10 mV (negative inside with regard to outside), as is clearly evident in Figure 14, Figure 15, Figure 16 and Figure 17. This large depolarization significantly increases the fraction of the inactivated sodium channels blocking the neuronal activity even though that membrane potential is depolarized, and this can be served to explain the inhibitory effects of anesthetic agents on consciousness.
5. Interestingly, as the quantum tunneling model predicts the ability of GABA receptor to permeate chloride ions without the binding of GABA ligand at certain range of gate energy, this comes consistent with the spontaneous activity of GABA receptors without the requirement of GABA binding, which has been observed and mentioned in the literature [39].

These proposed mechanisms require experimental verification to prove the scientific validity of the quantum tunneling model of chloride ions. Hence, the present study represents the first stage of the investigation process towards better understanding of the biological function of GABA receptors from the quantum mechanical perspective and towards more established quantum biology and quantum medicine.

## 5. Conclusions

The quantum tunneling model of chloride ions can explain the depolarization effect of GABA receptors at normal intracellular chloride ions without the requirement of the intracellular chloride ions accumulations. The quantum tunneling-induced membrane depolarization has been found to start at around *G* = 2 J.

## Figures and Tables

**Table 1:** The table represents the curving energy point, the membrane potential at *G* = 1 J, and the average rate of depolarization at different values of gate length L and according to the other setting values in Figure 14.

		L = 1 m	
Ion	*G_cur_*	*V_m_* at *G* = 1 J	*R*
Cl	2.35 J	0.0064 V	0.046 V/J
		L = 1.5 m	
Ion	*G_cur_*	*V_m_* at *G* = 1 J	*R*
Cl	1.98 J	0.0044 V	0.066 V/J
		L = 2 m	
Ion	*G_cur_*	*V_m_* at *G* = 1 J	*R*
Cl	1.8 J	0.0039 V	0.081 V/J

**Table 2:** The table represents the curving energy point, the membrane potential at *G* = 1 J, and the average rate of depolarization at different values of gate location *n* and according to the other setting values in Figure 15.

		*n* = 1	
Ion	*G_cur_*	*V_m_* at *G* = 1 J	*R*
Cl	1.2 J	0.0062 V	0.31 V/J
		*n* = 2	
Ion	*G_cur_*	*V_m_* at *G* = 1 J	*R*
Cl	1.98 J	0.0044 V	0.066 V/J
		*n* = 3	
Ion	*G_cur_*	*V_m_* at *G* = 1 J	*R*
Cl	2.23 J	0.004 V	0.053 V/J

**Table 3:** The table represents the curving energy point, the membrane potential at *G* = 1 J, and the average rate of depolarization at different values of channels density *D* and according to the other setting values in Figure 16.

		*D* = 10^9^ channels/cm^2^	
Ion	*G_cur_*	*V_m_* at *G* = 1 J	*R*
Cl	1.91 J	0.0044 V	0.071 V/J
		*D* = 10^10^ channels/cm^2^	
Ion	*G_cur_*	*V_m_* at *G* = 1 J	*R*
Cl	1.98 J	0.0044 V	0.066 V/J
		*D* = 10^11^ channels/cm^2^	
Ion	*G_cur_*	*V_m_* at *G* = 1 J	*R*
Cl	2.05 J	0.0044 V	0.062 V/J

## Data Availability

Not applicable.
